# Comparative performance of the GenoLab M and NovaSeq 6000 sequencing platforms for transcriptome and LncRNA analysis

**DOI:** 10.1186/s12864-021-08150-8

**Published:** 2021-11-17

**Authors:** Yongfeng Liu, Ran Han, Letian Zhou, Mingjie Luo, Lidong Zeng, Xiaochao Zhao, Yukun Ma, Zhiliang Zhou, Lei Sun

**Affiliations:** 1 GeneMind Biosciences Company Limited, ShenZhen, China; 2 Beijing Guoke Biotechnology Co., LTD, Beijing, China

**Keywords:** GenoLab M, NovaSeq 6000, Transcriptome, LncRNA, Compatibility

## Abstract

**Background:**

GenoLab M is a recently established next-generation sequencing platform from GeneMind Biosciences. Presently, Illumina sequencers are the globally leading sequencing platform in the next-generation sequencing market. Here, we present the first report to compare the transcriptome and LncRNA sequencing data of the GenoLab M sequencer to NovaSeq 6000 platform in various types of analysis.

**Results:**

We tested 16 libraries in three species using various library kits from different companies. We compared the data quality, genes expression, alternatively spliced (AS) events, single nucleotide polymorphism (SNP), and insertions–deletions (InDel) between two sequencing platforms. The data suggested that platforms have comparable sensitivity and accuracy in terms of quantification of gene expression levels with technical compatibility.

**Conclusions:**

Genolab M is a promising next-generation sequencing platform for transcriptomics and LncRNA studies with high performance at low costs.

**Supplementary Information:**

The online version contains supplementary material available at 10.1186/s12864-021-08150-8.

## Background

The past dozens of years have witnessed a new era in functional genomics using sequencing technologies [1]. The launch of the Roche 454 sequencer opened the era of next-generation sequencing (NGS) [2]. Compared with the traditional Sanger sequencing technology [3], NGS has significantly higher throughput and reduced costs [1]. Taking advantages of the power of NGS, transcriptome and Long non-coding RNA (LncRNA) sequencing has been accepted as a mainstream profiling technique to reveal gene regulatory networks in both animals and plants [4].

In the short history of NGS era, many sequencing platforms have emerged: Roche 454, Illumina series (GA, HiSeq, NextSeq, NovaSeq, etc.) [5], BGI (BGISEQ-500) [6], Ion Torrent [7], GenapSys [8]. These platforms employ different sequencing chemistry and detection approaches, and each of them has specific advantages and shortcomings [9]. After years of technology evolution and product commercialization, Illumina sequencers become the most widely used platform. However, the high instrument and reagent cost hinders broader applications [10]. In recent years, BGI’s MGI sequencers have received more attention in their cost effectiveness [11], though BGI’s unique DNB (DNA Nanoball) sequencing approach requires complicated library preparation and quality control procedure [12–15]. As DNA sequencing applications increase in different research fields and clinical settings, there is still a need to develop sequencers that are accurate, flexible, and cost-efficient for applications.

**Fig. 1:**
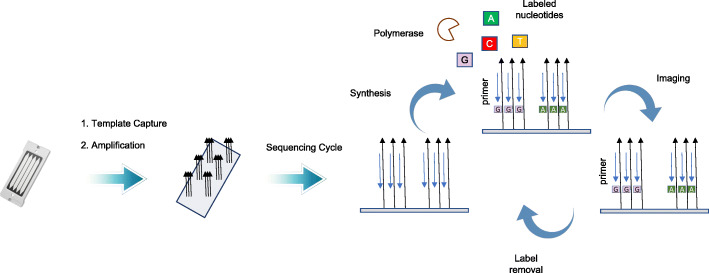
Sequencing Workflow of GenoLab M

Recently, GeneMind Biosciences Company Limited (GeneMind), launched a new sequencing instrument (GenoLab M™) based on their previous work on GenoCare™ single molecule sequencer [16]. An overview of the mechanism of GenoLab M DNA sequencer is outlined in Fig. 1. The GenoLab M sequencer employs sequencing-by-synthesis (SBS) techniques and applies reversible termination approaches. In a sequencing run, a double-stranded target DNA library is constructed with generic adaptor sequences. The library is denatured to create single-stranded templates, which are captured on the surface of flow cell through hybridization to randomly pre-immobilized complimentary oligonucleotide. Surface-based amplification is performed after target DNA template capture to enhance signal-to-noise ratio of sequencing. The amplified DNA colonies on the flow cell are then hybridized to a sequencing primer, which contains an adaptor-complimentary sequence. Next, Fluorescence-dye labeled nucleotides and a polymerase are applied to start the sequencing cycle. In each cycle, the nucleotides’ terminator structure ensures only one nucleotide is incorporated by the polymerase on each extending primer. Four-color fluorescence signals from the labels are collected by a scanning optical system, and the terminator structure is cleaved to initiate the next sequencing cycle. The fluorescence image data through all cycles are then combined and color-corrected to generate the raw basecall data. Finally, Sequencing quality score are assigned to each base, DNA reads with the corresponding quality scores are combined to produce the final fastq file.

NovaSeq 6000, launched in June, 2017, relies on Illumina’s SBS chemistry and two-color reversible terminator-based method. Combined with patterned flow cell technology [17], in excess of 3000 Gb of data can be sequenced on an S4 flow cell.

Previously, GenoLab M’s performance on transcriptome and LncRNA has not yet been evaluated by the scientific community. Here, we characterized the performance of GenoLab M on transcriptome and LncRNA by parallel comparison with NovaSeq 6000 from Illumina, Inc. on three different species: mouse, bean, and human. The raw data quality, gene expression level, alternatively spliced (AS) events, single nucleotide polymorphism (SNP), and insertions–deletions (InDel) analysis from the two sequencing platforms are compared. The data suggest that the GenoLab M is a promising sequencing platform for transcriptomics and LncRNA studies in animal, plant and human with comparable performance at lower cost.

## Method

### Samples preparation and RNA extraction

Mouse testicular tissue, human Lieming Xu-2 cells and bean hairy root tissue were collected for RNA extraction. RNAs were extracted by HiPure Universal RNA Mini Kit (Guangzhou Magen Biotechnology Co., Ltd.). Total RNA concentration and purity and integrity were measured via NanoDrop 2000 (Thermo Fisher Scientific, Wilmington, DE) and RNA Nano 6000 Assay Kit of the Agilent Bioanalyzer 2100 system (Agilent Technologies, CA, USA), respectively.

### Transcriptome and LncRNA sequencing

Transcriptome library construction were performed by Hieff NGS Ultima Dual-mode mRNA Library Prep Kit for Illumina (Yeasen Biotechnology (Shanghai) Co., Ltd., China), Fast RNA-seq Lib Prep Module for Illumina (ABclonal Technology Co.,Ltd., China), TIANSeq Stranded RNA-Seq Kit (Illumina) (TIANGEN Biotech (Beijing) Co., Ltd., China) and VAHTS Universal V6 RNA-seq Library Prep Kit for Illumina (Vazyme Biotech Co., Ltd., China). These mRNA libraries were marked as Mouse, Human or Bean_mRNA_YS, AB, TG or VZ. LncRNA library were constructed via Hieff NGS Ultima Dual-mode RNA Library Prep Kit for Illumina and Hieff NGS MaxUp rRNA Depletion Kit (human/mouse/rat) (Yeasen Biotechnology (Shanghai) Co., Ltd., China), VAHTS Universal V6 RNA-seq Library Prep Kit for Illumina and Ribo-off rRNA Depletion Kit (Human/Mouse/Rat) (Vazyme Biotech Co.,Ltd., China), TIANSeq Stranded RNA-Seq Kit (Illumina) and TIANSeq rRNA Depletion Kit (H/M/R) (NR101-TA) (TIANGEN Biotech (Beijing) Co.,Ltd.,China). These libraries were marked as Mouse or Human_LncRNA_YS, VZ or TG. After library QC, they were subjected to NovaSeq 6000 and GenoLab M sequencing in PE150 or PE100 mode.

### Cross-platform mRNA and LncRNA sequencing data analysis

Raw sequencing reads in fastq format were processed through a GeneMind in-house perl pipeline. Reads containing adapter, ploy-N or low-quality reads were filtered out to get clean reads. These clean reads were then mapped to the reference genome sequence with a perfect match or one mismatch method via HISAT2 tools software [18]. The corresponding genome references were downloaded from ensemble database by ftp://ftp.ensembl.org/pub/release-101/fasta/homo_sapiens/dna/, ftp://ftp.ensembl.org/pub/release-101/fasta/mus_musculus/dna, and ftp://ftp.ensemblgenomes.org/pub/plants/release-48/fasta/glycine_max/dna/. StringTie [19] was then used for transcript reconstruction. As candidate genes were defined as genes which were mapping to unannotated transcribed region, meanwhile, coding peptide was more than 50 amino acid residues with two or more exons. SNP and InDel calling was carried out by using GATK [20], furthermore, SnpEff [21] was used to annotate these mutations. Raw vcf files were filtered with GATK standard filter method and other parameters (clusterWindowSize:10; MQ0 > = 4 and (MQ0/(1.0*DP)) > 0.1; QUAL < 10; QUAL < 30.0 or QD < 5.0 or HRun > 5), and only SNPs with distance > 5 were retained. Alternative spliced events were identified by ASprofle software [22]. Expression values of candidate genes (FPKM) were calculated by RSEM [23].

For LncRNA identification, bioinformatic pipeline was performed according to published methods [24] with minor modifications. The transcriptome was assembled using the StringTie based on the reads mapped to the reference genome. The known LncRNAs from the assembled transcripts are defined using the Cuffcompare program from the Cufflinks package. The remaining transcripts (unknown transcripts) were used to screen for putative LncRNAs. Transcripts of more than 200 nt length and two exons were selected as candidate LncRNA transcripts. Then, CPC [25], CNCI [26], Pfam [27] and CPAT [28] were used to distinguish the protein-coding genes from the non-coding genes, and inter set as putative LncRNA. As well as the different types of LncRNAs including lincRNA, intronic LncRNA, anti-sense LncRNA, sense LncRNA were selected using gffcompare. StringTie (1.3.1) [29] was used to calculate FPKMs of LncRNAs. The FPKM of novel LncRNAs must be ≥0.1.

## Results

### Base and raw data quality

**Fig. 2:**
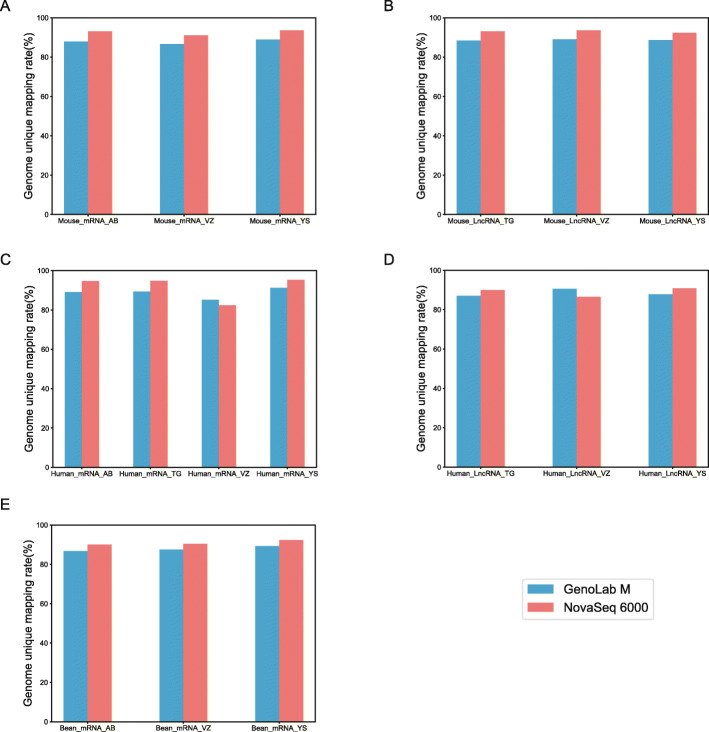
Comparison of sequencing quality between GenoLab M and NovaSeq 6000 in genome mapping rate. **A** Transcriptome of mouse, **B** LncRNA of mouse, **C** Transcriptome of human, **D** LncRNA of human, **E** Transcriptome of bean. AB_, VZ_, YS_,TG_ means library kits from four companies

**Fig. 3:**
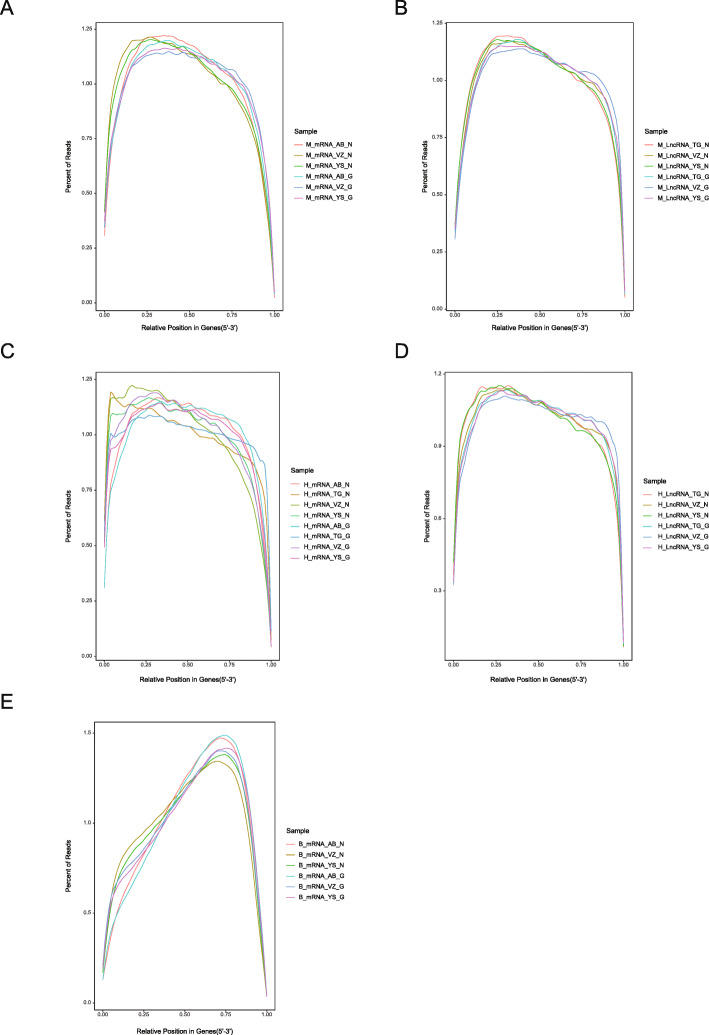
Comparison of sequencing quality between GenoLab M and NovaSeq 6000 in Reads distribution along the relative position of genes. **A** Transcriptome of mouse, **B** LncRNA of mouse, **C** Transcriptome of human, **D** LncRNA of human, **E** Transcriptome of bean. M_, H_ and B_ means mouse, human and bean, AB_, VZ_, YS, TG_ means library kits from four companies, N and G means Novaseq 6000 and GenoLab M

**Fig. 4:**
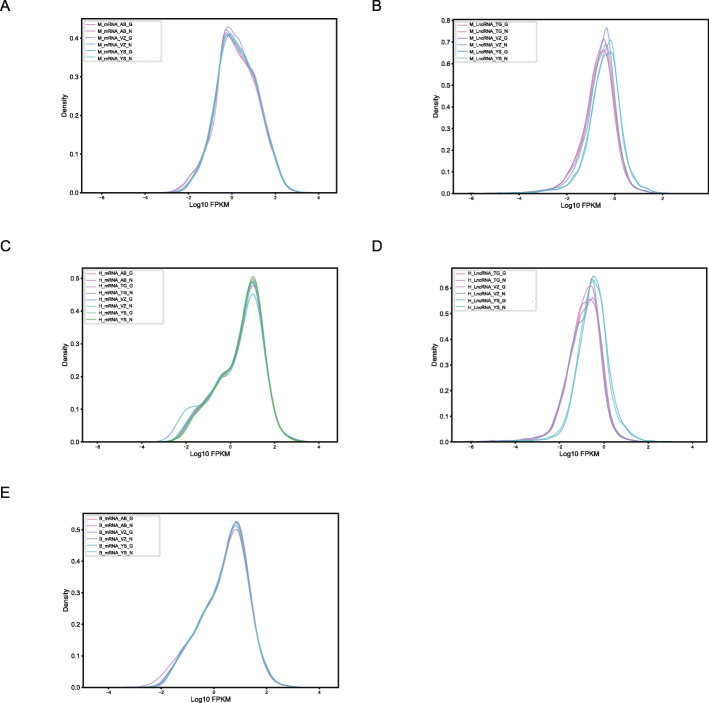
Repeatability of gene detection and quantifcation between GenoLab M and NovaSeq 6000 in expression density distribution. **A** Transcriptome of mouse, **B** LncRNA of mouse, **C** Transcriptome of human, **D** LncRNA of human, **E** Transcriptome of bean. M_, H_ and B_ means mouse, human and bean, AB_, VZ_, YS, TG_ means library kits from four companies, N and G means Novaseq 6000 and GenoLab M

**Fig. 5:**
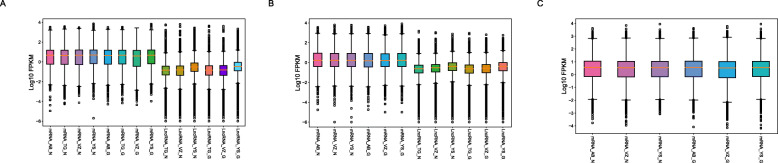
Gene and lncRNA detection and quantifcation between GenoLab M and NovaSeq 6000 in boxplot graph of **A** human, **B** mouse, **C** bean. AB_, VZ_, YS_,TG_ means library kits from four companies, N and G means Novaseq 6000 and GenoLab M

Following RNA extraction, two aliquots of each extract were constructed as Illumina libraries, respectively, using identical amounts of starting material, and then subsequently sequenced to facilitate bioinformatic comparisons on the data. In addition, to verify the compatibility of the library preparation kit for GenoLab M, we used kits from different manufacturers for testing (Supplemental Table S1). The sequencing strategy was pair-end 100 bp for GenoLab M and paired-end 150 bp for NovaSeq 6000. We initially generated between 23.20 M to 62.87 M clean reads per library in NovaSeq 6000 platform, and 26.86 M to 139.69 M clean reads per library in GenoLab M platform (Table 1). Each individual sample has similar base throughput from both sequencing platforms. The quality of sequencing data was checked using FastQC. For high base quality (over Q20) base percentages, the GenoLab M showed an average of 94.86%, and the NovaSeq 6000 showed an average of 97.50% with a slight preponderance (Table 1). As shown in Fig. 2, the clean reads from GenoLab M reached an average mapping rate of 91.80% and an average unique mapping rate of 88.33%, which are comparable to the mapping rates of reads from the NovaSeq 6000 platform. The two platforms shared fairly consistent reads distribution along genes across species (Fig. 3) and in expression density distribution (Fig. 4). Interestingly, the LncRNA expression level measured using Yeasen LncRNA library kit (YS) is higher than the other kits used in human and mouse. In Fig. 5, the charts showed that accuracy in the quantification of both low and high abundance genes were consistent. They further indicate that LncRNA expression by YS has obviously higher abundancy than the other kits in human and mouse (Fig. 5 A and B), which is consistent with the Fig. 4 B and D. Overall, the sequence quality of the two platforms was similar across various library kits.

**Table 1:** Summary of basic parameters in six transcriptome and four LncRNA sequencing datasets

Sample ID	Species	RNA Type	GenoLab M (PE100)			NovaSeq 6000 (PE150)		
			Reads (M)	Bases (Gb)	Q20(%)	Reads (M)	Bases (Gb)	Q20(%)
T1–3301-2-AB	Bean	Transcriptome	33.75	6.74	95.65	24.67	7.36	97.45
T1–3301-2-VZ	Bean	Transcriptome	139.69	27.81	94.25	38.55	11.40	97.33
T1–3301-2-YS	Bean	Transcriptome	26.86	5.36	95.90	23.20	6.91	97.39
T1-TGF-AB	Human	Transcriptome	33.54	6.70	95.06	25.36	7.58	97.64
T1-TGF-TG	Human	Transcriptome	29.30	5.86	94.31	30.29	9.06	97.58
T1-TGF-VZ	Human	Transcriptome	79.46	15.85	93.49	33.49	9.88	97.54
T1-TGF-YS	Human	Transcriptome	31.10	6.21	95.35	24.09	7.20	97.62
T1-C1-AB	Mouse	Transcriptome	50.48	10.08	94.04	26.30	7.86	97.52
T1-C1-VZ	Mouse	Transcriptome	78.00	15.55	93.24	36.78	10.96	97.26
T1-C1-YS	Mouse	Transcriptome	27.57	5.51	94.74	24.20	7.23	97.73
T2-TGF-TG	Human	LncRNA	59.77	11.94	95.07	44.71	13.27	97.37
T2-TGF-VZ	Human	LncRNA	64.01	12.79	95.63	62.87	18.67	97.95
T2-TGF-YS	Human	LncRNA	36.74	7.34	94.89	32.83	9.79	97.35
T2-C1-TG	Mouse	LncRNA	69.69	13.92	95.05	50.33	14.94	97.32
T2-C1-VZ	Mouse	LncRNA	69.91	13.97	95.52	39.80	11.90	97.42
T2-C1-YS	Mouse	LncRNA	52.03	10.39	95.58	34.40	10.25	97.57

### Inter-platforms comparison of gene detection and quantification

**Fig. 6:**
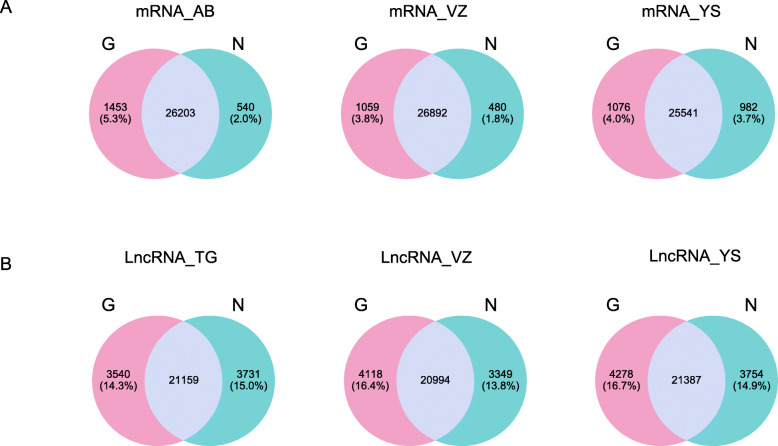
Venn diagram of genes expression FPKM between GenoLab M and NovaSeq 6000 in mouse. **A** Transcriptome, **B** LncRNA. AB_, VZ_, YS_,TG_ means library kits from four companies, N and G means Novaseq 6000 and GenoLab M

**Fig. 7:**
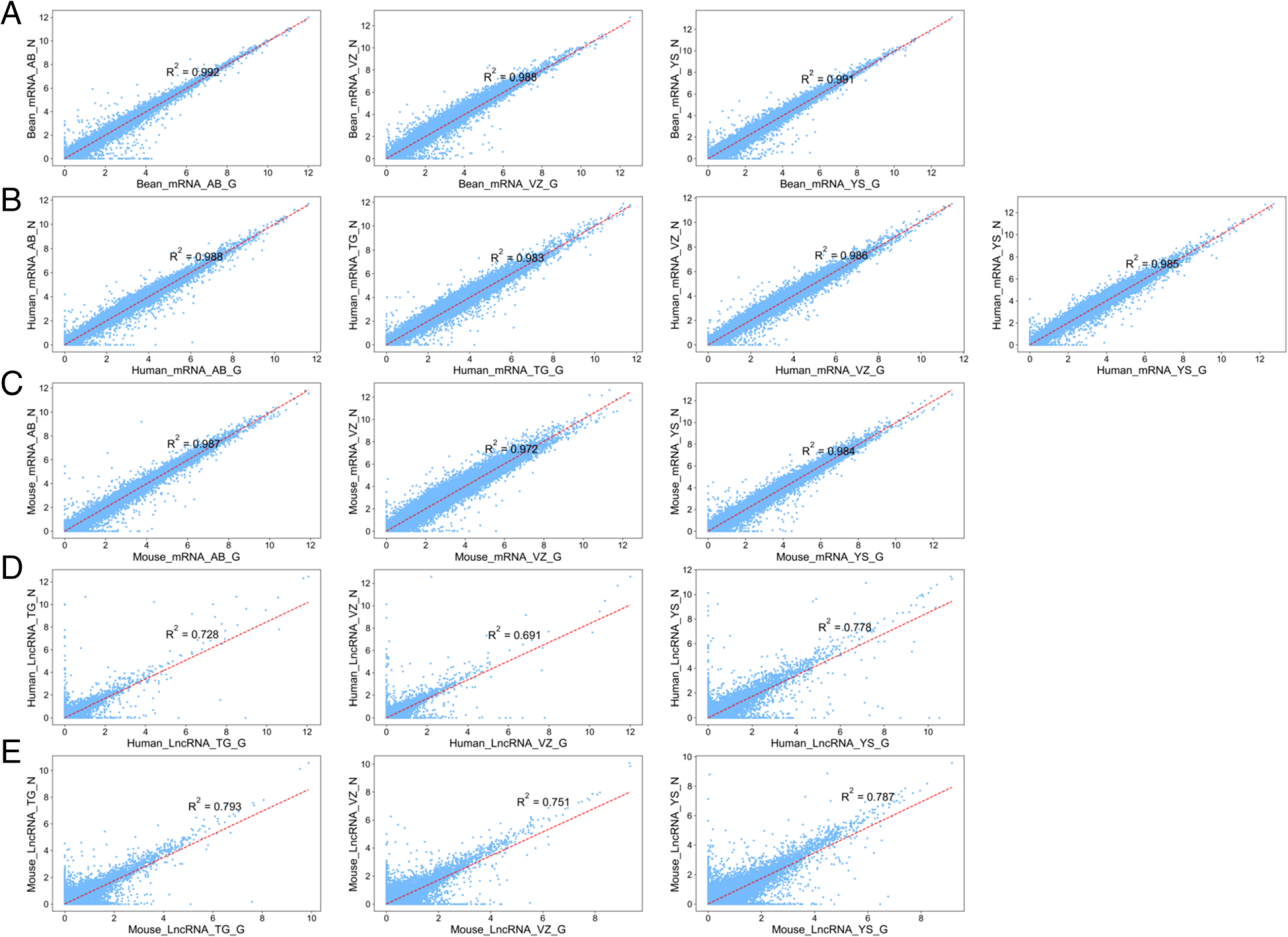
Scatter plots of gene expression values of the four pairs of samples produced using the NovaSeq 6000 and GenoLab M sequencers. Gene expression values are represented as the base 2 logarithm of FPKM. The Pearson correlation coefficients of the 16 samples were between 0.69 and 0.99. **A** Transcriptome of bean, **B** Transcriptome of human, **C** Transcriptome of mouse, **D** LncRNA of human, E LncRNA of mouse

In transcriptome and LncRNA analysis, the identification of genes is very important for the majority of research projects. Therefore, we further compared the capacity of GenoLab M and NovaSeq 6000 platforms on gene detection and quantification. Totally over 42,000, 16,000 and 26,000 genes were identified in bean, human, and mouse, respectively, via two sequencing platforms (Fig. 6A, Fig.S1A&B). For transcriptome, we observed a small fraction of different genes between the GenoLab M and NovaSeq 6000 platforms. Over 92% of genes were commonly detected by both sequencing platforms. However, for LncRNA, only 71% of genes were shared between the two sequencing platforms (Fig. 6B, Fig. S1C). This difference most likely stemmed from analysis using the method StringTie as novel LncRNAs judgment and the different read length of the sequence [19]. StringTie (1.3.1) was used to calculate FPKMs of LncRNAs and novel LncRNA was set at least 0.1. We checked the Pearson correlation coefficient of the transcriptome and LncRNA data produced by the two platforms using the same methods and found that all one pairs of samples showed high correlation coefficients, ranging from 0.972 to 0.992 in transcriptome, and ranging from 0.691 to 0.793 in LncRNA (Fig. 7). There is still a slight gap in the correlation between LncRNA and the two platforms. In all, GenoLab M has remarkable inter-platforms concordance with NovaSeq 6000, suggesting that GenoLab M could substitute NovaSeq 6000 in many application fields where transcriptome and LncRNA are the primary focus.

### Detection of alternative splicing

**Fig. 8:**
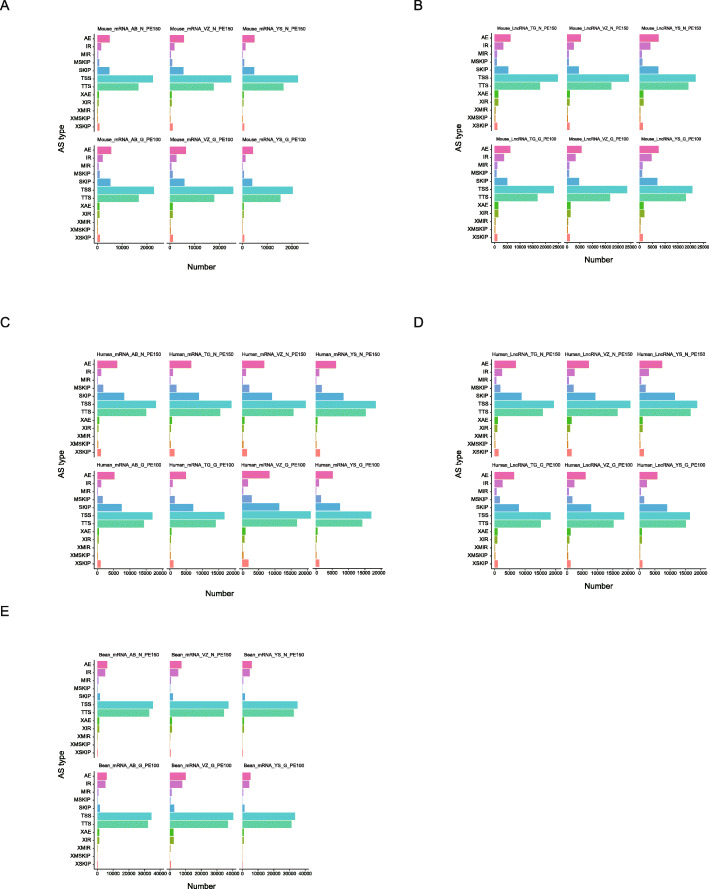
Alternative splicing events of mRNA and lncRNA FPKM between GenoLab M and NovaSeq 6000. **A** Transcriptome of bean, **B** Transcriptome of human, **C** Transcriptome of mouse, **D** LncRNA of human, **E** LncRNA of mouse. TSS: Alternative 5′ first exon (transcription start site), TTS: Alternative 3′ last exon (transcription terminal site), SKIP: Skipped exon (SKIP_ON,SKIP_OFF pair), XSKIP: Approximate SKIP (XSKIP_ON,XSKIP_OFF pair), MSKIP: Multi-exon SKIP (MSKIP_ON,MSKIP_OFF pair), XMSKIP: Approximate MSKIP (XMSKIP_ON,XMSKIP_OFF pair), IR: Intron retention (IR_ON, IR_OFF pair), XIR: Approximate IR (XIR_ON,XIR_OFF pair), MIR: Multi-IR (MIR_ON, MIR_OFF pair), XMIR: Approximate MIR (XMIR_ON, XMIR_OFF pair), AE: Alternative exon ends (5′, 3′, or both), XAE: Approximate AE

As one of the major mechanisms to generate transcriptome diversity, alternative splicing (AS) is gaining more and more attention in recent years. In this context, the ability of each sequencing platform under comparison to detect splicing junctions and corresponding alternative splicing patterns were subsequently analyzed across transcriptomes. In mouse, 53,557, 59,709 and 53,014, 56,741, 64,105 and 48,089 AS events could be detected by GenoLab M and NovaSeq 6000, respectively. Top three AS events in all libraries were TSS: Alternative 5′ first exon (transcription start site), TTS: Alternative 3′ last exon (transcription terminal site) and AE: Alternative exon ends (5′, 3′, or both) cross two platforms (Fig. 8 A). In mouse LncRNA data, the AS events component in mRNA presented similarly to transcriptome (Fig. 8 B). For human sample, AS events component in transcriptome and mRNA of LncRNA data were of the same pattern and Top 3 AS were TSS, TTS and SKIP:Skipped exon (SKIP_ON,SKIP_OFF pair) as showed in Fig. 8 C and D. In beans, 78,137, 82,558 and 105,038, 83,072, 84,526 and 90,580 AS events could be detected by GenoLab M and NovaSeq 6000, respectively. Top three AS events in all libraries were TSS, TTS and AE (Fig. 8 E). As for both the number and the type of different AS events, we found that there was no significant difference between the three species in the two platforms.

### Identification of SNP and InDel mutation

SNP and InDel are crucial genomic features to reveal genetic variation. High throughput transcriptome analysis contributes to how these DNA variations can be transcribed into RNA messengers to affect subsequent protein function. Therefore, we examined the competency of the GenoLab M sequencing platform to detect SNP and InDel variations at the mRNA level. Regarding SNP detection, we found that SNPs called from the two sequencing platforms (Table 2) were highly similar in both variety and quantity. The largest difference is that the GenoLab M platform identified slightly more SNP events in mice than NovaSeq 6000 on average.

**Table 2:** Summary of SNP identifcation in all samples

Sample	A- > G	G- > A	C- > T	T- > C	Transition	A- > C	C- > A	A- > T	T- > A	C- > G	G- > C	G- > T	T- > G	Transversion	Total
Bean_mRNA_AB_N	13,754	13,281	13,327	13,609	53,971	4549	4512	6766	6591	3330	3342	4450	4538	38,078	92,049
Bean_mRNA_VZ_N	13,260	12,819	12,938	13,308	52,325	4381	4402	6479	6294	3260	3277	4268	4414	36,775	89,100
Bean_mRNA_YS_N	13,360	12,847	12,998	13,244	52,449	4339	4409	6552	6401	3268	3275	4336	4472	37,052	89,501
Bean_mRNA_AB_G	13,267	12,816	12,876	13,047	52,006	4357	4425	6772	6430	3213	3214	4337	4416	37,164	89,170
Bean_mRNA_VZ_G	15,421	14,791	14,768	15,323	60,303	4991	5084	7545	7311	3787	3796	4997	5031	42,542	102,845
Bean_mRNA_YS_G	12,393	11,956	11,975	12,390	48,714	4050	4093	6223	6012	3024	2989	4050	4056	34,497	83,211
Human_mRNA_AB_N	28,147	19,847	19,745	27,765	95,504	4212	4279	3205	3296	5208	5153	4161	4283	33,797	129,301
Human_mRNA_TG_N	26,667	19,444	19,386	26,452	91,949	4069	4152	3028	3060	5298	5168	4101	4049	32,925	124,874
Human_mRNA_VZ_N	23,206	18,098	17,877	23,211	82,392	3825	3865	2824	2803	5035	4889	3830	3850	30,921	113,313
Human_mRNA_YS_N	18,333	15,280	15,253	18,499	67,365	3134	3255	2322	2279	4172	4038	3204	3199	25,603	92,968
Human_mRNA_AB_G	22,677	18,164	17,955	22,700	81,496	3785	3797	3340	3365	4702	4605	3799	3840	31,233	112,729
Human_mRNA_TG_G	17,891	14,885	14,971	17,824	65,571	3101	3146	2778	2740	3963	3825	3080	3164	25,797	91,368
Human_mRNA_VZ_G	28,066	23,315	23,117	28,012	102,510	4978	5198	4612	4653	6187	6016	5126	5004	41,774	144,284
Human_mRNA_YS_G	16,090	14,008	13,905	16,040	60,043	2971	3000	2509	2466	3760	3681	2886	3027	24,300	84,343
Mouse_mRNA_AB_N	1868	1186	1205	1833	6092	342	386	428	460	362	377	372	340	3067	9159
Mouse_mRNA_VZ_N	2612	1416	1484	2582	8094	424	448	548	531	403	371	457	408	3590	11,684
Mouse_mRNA_YS_N	1998	1311	1263	1989	6561	346	400	387	401	312	274	363	314	2797	9358
Mouse_mRNA_AB_G	2252	1328	1402	2100	7082	428	456	1069	1025	381	379	439	400	4577	11,659
Mouse_mRNA_VZ_G	2871	1721	1687	2778	9057	620	809	1189	1109	538	524	818	632	6239	15,296
Mouse_mRNA_YS_G	2054	1063	1046	1902	6065	296	253	711	719	273	268	291	270	3081	9146

*Note*: A- > G means base A mutation as base G, Transition are interchanges of two-ring purines (A- > G and G- > A) or of one-ring pyrimidines (C- > T and T- > C), Transversions are interchanges of purine for pyrimidine bases, which therefore involve exchange of one-ring and two-ring structures. AB_, VZ_, YS_,TG_ means library kits from four companies, N and G means Novaseq 6000 and GenoLab M

For InDel events, GenoLab M detected less of them than the NovaSeq 6000 in bean, human and mouse (Table 3). The closest InDel number was in bean sample prepared with Vazyme Biotech (VZ) transcriptome library kit, while significant difference was observed in mouse via Yeasen Biotechnology (YS) transcriptome library kit. These results suggest that GenoLab M has slightly inferior in InDel detection, probably due to shorter read length in this study.

**Table 3:** Summary of InDel identifcation and effect type

Sample	Intergenic	Intron	Upstream	Downstream	CDS	Other	Total
Bean_mRNA_AB_N	117	2609	4131	3784	936	5914	17,491
Bean_mRNA_VZ_N	94	2363	3972	3406	889	5667	16,391
Bean_mRNA_YS_N	92	2116	3578	3096	727	5150	14,759
Bean_mRNA_AB_G	76	1887	3078	2770	663	4593	13,067
Bean_mRNA_VZ_G	102	2172	3656	3079	730	5162	14,901
Bean_mRNA_YS_G	57	1328	2370	2163	486	3878	10,282
Human_mRNA_AB_N	569	6704	1402	3529	112	5243	17,559
Human_mRNA_TG_N	598	6378	1597	3737	142	5592	18,044
Human_mRNA_VZ_N	607	5636	1376	3177	121	4882	15,799
Human_mRNA_YS_N	310	3578	1110	2628	73	4296	11,995
Human_mRNA_AB_G	334	3836	779	2336	71	3817	11,173
Human_mRNA_TG_G	248	2705	645	1973	78	3523	9172
Human_mRNA_VZ_G	459	4929	1125	2743	91	4217	13,564
Human_mRNA_YS_G	194	2062	605	1713	53	3099	7726
Mouse_mRNA_AB_N	480	2323	846	786	23	1866	6324
Mouse_mRNA_VZ_N	728	3194	1124	1012	35	2320	8413
Mouse_mRNA_YS_N	381	1444	640	581	18	1507	4571
Mouse_mRNA_AB_G	295	1385	519	532	16	1299	4046
Mouse_mRNA_VZ_G	415	1752	606	607	26	1544	4950
Mouse_mRNA_YS_G	119	533	257	278	7	694	1888

*Note*: CDS contains Start Lost, Frame Shift, Codon Deletion, Codon Insertion, Codon Change Plus Codon Deletion, Codon Change Plus Codon Insertion, and Stop Gained.AB_, VZ_, YS_,TG_ means library kits from four companies, N and G means Novaseq 6000 and GenoLab M

## Discussion

With the advantages of high-throughput and low cost, NGS is becoming a powerful tool for scientific and clinical research. Increased sequencing accessibility and flexibility have not only broadened NGS applications, but also led to the development of novel sequencing platforms and sequencing methods in turn [30]. Currently, Illumina’s sequencers are the globally leading sequencing platform. The NovaSeq 6000, its most powerful instrument, has prominent properties of lower error rate and less variation compared to other sequencers in the Illumina series [31]. It is able to generate 6 TB of sequencing data in a single run with a running cost between 12 and 18 USD/Gb [32]. GenoLab M, the new sequencer of GeneMind, can generate 300 Gb of sequence data in a single run with price per Gb cost less than half of that. In this study, we generated large transcript and LncRNA datasets from the two sequencing platforms across three model species (human: 4 mRNA, 3 LncRNA; mouse: 3 mRNA, 3 LncRNA; bean: 3 mRNA). Next, we compared the datasets obtained from the two platforms. To make our study as comprehensive as possible, we compared the quality of data, distribution of reads, gene expression, AS, SNP and InDel of the two platforms.

Our analysis of the data generated from two platforms showed that sequences from both instruments were of comparable quality with the exception that NovaSeq 6000 reads showed slightly higher Q20 percentage than GenoLab M. We are confident that higher quality data from GenoLab M are attainable through instrument hardware, software and reagent kit updates, given that the instrument was launched just last year [33, 34].

Gene expression has always been an important part of the research on transcriptome and LncRNA [34]. In the comparison of transcriptome genes expression, we found that there was no significant quality difference between the two platforms, and the correlation analysis showed high consistency. This indicates that GenoLab M can achieve a similar level of mRNA detection as NovaSeq 6000 and is suitable for use with the same library kits designed for Illumina sequencing. This compatibility enables users to test the sequencing platform with minimum transition cost and generate high quality sequencing data. We believe that this would make transcriptome sequencing more accessible for researchers. However, in the LncRNA area, we found that GenoLab M’s performance had a small gap compared to NovaSeq 6000 in terms of gene expression correlation. We think sequencing read length may impact the LncRNA detection rate [35]. We plan to conduct further laboratory tests to determine the cause of this difference.

Our experimental results proved that GenoLab M could obtain equivalent data quality as NovaSeq 6000, in both mRNA and LncRNA level with 7 library preparation kits from 4 companies. This suggests that GenoLab M can be a viable substitute for NovaSeq 6000 in the RNA sequencing. However, our study does lack biological repeats, which could be supplemented in further work. We also realize that we still need to increase the number of samples and species to further demonstrate the reliability of the GenoLab M platform. In the future, we plan to work with more researchers in broader application areas to verify the capability and stability of the platform.

## Conclusions

In summary, we highlight that both GenoLab M and NovaSeq 6000 sequencing platforms have similar and comparable performance metrics (sensitivity and accuracy) and can capture genes, AS, and SNP at transcriptome and LncRNA levels. The GenoLab M offers a cost-effective alternative to the NovaSeq 6000 platform with similar data quality.

## Supplementary Information

**Additional file 1.**

## Data Availability

The transcriptome and LncRNA data are in available at SRA database with SUB10177917 and SUB10176628, and can be downloaded by extract Code GMData in https://e-share.obs-website.cn-north-1.myhuaweicloud.com?token=qWLThbSnZgWWLy6t2a4fclhufssdkZDe/3/fC3VlxxVMb566Lbq4r+BTnK09LdfBGM7oGcHDf7zTbsEslDLqt15oyX4jumktMD5vQWxNtSeFF4qb2g1tvvZ02xi49VXUV2VcMW5MjekUW86kgWa4XaF0wAhw1fmDnUMGoI3MMb/iPkwj7gOaFNQ0RvRNBQbO0l1xVdor34j4jspNoiPfjI845l8MOe6nWbasiSUpWW6fpfLsTKWZxk30fnaWZe3Q9oMOu8h2abZbXyyhf4DhtYLZvjTM9K5jCGrERBA5LWjjyEYaZTx9/4QKf0Gy1iXGk9w/61Ub20trdgeliFgGrYN/jr9QOngRpdZWJ/sYaLSsAgWuU94R7SdjygvdZV4hViwlltsRcHUGArhnVqMe+uUg5Eb7O6X4fUCTz2AUdhMxsZBmuF2xjHAaV5EcvRZPkvw7+2EZ+nmfefxYCRBJgyP5Rfr7wMpUiuf/NsOIynyXxbJlHcN/ByYdkRdkWywk9/2w1YCpv4kNYHchhvC+gXB+NbRMByg3bQxT24G9jw430ynyN3P4+z9G/e78/XEEqJXK/9Z/BNbA56sOiT6RsA==. The transcriptome and LncRNA data are deposited at the CNGB Sequence Archive (https://db.cngb.org/cnsa/) under project accession number CNP0002262.
