# Correction to: Comparative performance of the GenoLab M and NovaSeq 6000 sequencing platforms for transcriptome and LncRNA analysis

**DOI:** 10.1186/s12864-022-08307-z

**Published:** 2022-01-26

**Authors:** Yongfeng Liu, Ran Han, Letian Zhou, Mingjie Luo, Lidong Zeng, Xiaochao Zhao, Yukun Ma, Zhiliang Zhou, Lei Sun

**Affiliations:** 1 GeneMind Biosciences Company Limited, ShenZhen, China; 2 Beijing Guoke Biotechnology Co., LTD, Beijing, China

**Correction to: BMC Genomics 22, 829 (2021)**

**https://doi.org/10.1186/s12864-021-08150-8**

Following publication of the original article [1], it was reported that the article erroneously stated that the launch year of NovaSeq 6000 was 2013 instead of 2017.

Furthermore, the following ‘Availability of data and materials’ and ‘Competing interests’ declarations have been added to the article:

**Availability of data and materials**

The transcriptome and LncRNA data are deposited at the CNGB Sequence Archive (https://db.cngb.org/cnsa/) under project accession number CNP0002262.

**Competing interests**

Lei Sun, Yongfeng Liu, XiaoChao Zhao, Zhiliang Zhou, Mingjie Luo, Letian Zhou, and Lidong Zeng are employees of GeneMind Biosciences Company Limited. All other authors declare that they have no competing interests.

The original article [1] has been updated.
