# Supplementary material for: Comparative performance of the GenoLab M and NovaSeq 6000 sequencing platforms for transcriptome and LncRNA analysis
Source: BMC Genomics. 2021 Nov 17;22:829. doi: 10.1186/s12864-021-08150-8 (PMC8600837; doi:10.1186/s12864-021-08150-8)
Supplement: Supplementary file 1 — Additional file 1. [file 12864_2021_8150_MOESM1_ESM.docx]

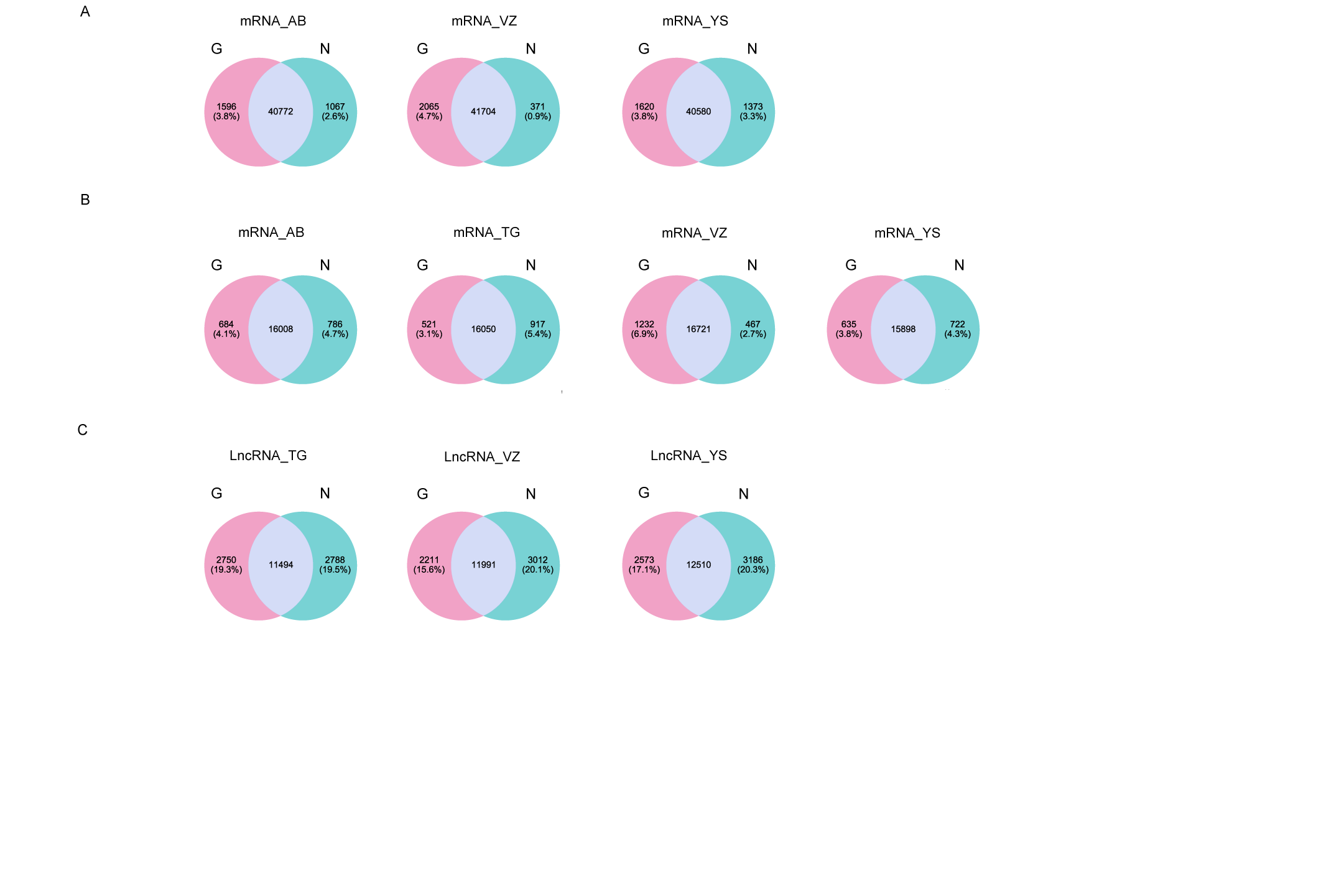


Figure S1 Venn diagram of genes expression FPKM between GenoLab M and NovaSeq 6000 in A Transcriptome of bean, B Transcriptome of human, C LncRNA of human. AB_, VZ_, YS_,TG_ means library kits from four companies.

| Sequencing type | Tissue | sample name | Kit reagent manufacturer | Kit Name | cargo no. |
| --- | --- | --- | --- | --- | --- |
| mRNA | mouse testicular tissu | lib-T1-C1-YS | Yeasen Biotechnology (Shanghai) Co., Ltd，China | Hieff NGS Ultima Dual-mode mRNA Library Prep Kit for Illumina | 12301 |
| mRNA | human cell line | lib-T1-TGF-YS | Yeasen Biotechnology (Shanghai) Co., Ltd，China | Hieff NGS Ultima Dual-mode mRNA Library Prep Kit for Illumina | 12301 |
| mRNA | soybean hairy root | lib-T1-3301.2-YS | Yeasen Biotechnology (Shanghai) Co., Ltd，China | Hieff NGS Ultima Dual-mode mRNA Library Prep Kit for Illumina | 12301 |
| mRNA | mouse testicular tissu | lib-T1-C1-AB | ABclonal Technology Co.,Ltd，China | Fast RNA-seq Lib Prep Module for Illumina | RK20304 |
| mRNA | human cell line | lib-T1-TGF-AB | ABclonal Technology Co.,Ltd，China | Fast RNA-seq Lib Prep Module for Illumina | RK20304 |
| mRNA | soybean hairy root | lib-T1-3301.2-AB | ABclonal Technology Co.,Ltd，China | Fast RNA-seq Lib Prep Module for Illumina | RK20304 |
| mRNA | human cell line | lib-T1-TGF-TG | TIANGEN Biotech(Beijing)Co.,Ltd,China | TIANSeq Stranded RNA-Seq Kit(Illumina) | NR103 |
| mRNA | mouse testicular tissu | lib-T1-C1-VZ | Vazyme Biotech Co., Ltd,China | VAHTS Universal V6 RNA-seq Library Prep Kit for Illumina | NR604-01/02 |
| mRNA | human cell line | lib-T1-TGF-VZ | Vazyme Biotech Co., Ltd,China | VAHTS Universal V6 RNA-seq Library Prep Kit for Illumina | NR604-01/02 |
| mRNA | soybean hairy root | lib-T1-3301.2-VZ | Vazyme Biotech Co., Ltd,China | VAHTS Universal V6 RNA-seq Library Prep Kit for Illumina | NR604-01/02 |
| lncRNA | mouse testicular tissu | lib-T2-C1-YS | Yeasen Biotechnology (Shanghai) Co., Ltd，China | Hieff NGS Ultima Dual-mode RNA Library Prep Kit for Illumina（12252）+Hieff NGS MaxUp rRNA Depletion Kit（human/mouse/rat）（12253） | 12252+12253 |
| lncRNA | human cell line | lib-T2-TGF-YS | Yeasen Biotechnology (Shanghai) Co., Ltd，China | Hieff NGS Ultima Dual-mode RNA Library Prep Kit for Illumina（12252）+Hieff NGS MaxUp rRNA Depletion Kit（human/mouse/rat）（12253） | 12252+12253 |
| lncRNA | mouse testicular tissu | lib-T2-C1-VZ | Vazyme Biotech Co., Ltd,China | VAHTS Universal V6 RNA-seq Library Prep Kit for Illumina(NR604-01/02)+Ribo-off rRNA Depletion Kit (Human/Mouse/Rat)(N406-01/02) | NR604-01/02+N406-01/02 |
| lncRNA | human cell line | lib-T2-TGF-VZ | Vazyme Biotech Co., Ltd,China | VAHTS Universal V6 RNA-seq Library Prep Kit for Illumina(NR604-01/02)+Ribo-off rRNA Depletion Kit (Human/Mouse/Rat)(N406-01/02) | NR604-01/02+N406-01/02 |
| lncRNA | mouse testicular tissu | lib-T2-C1-TG | TIANGEN Biotech(Beijing)Co.,Ltd,China | TIANSeq Stranded RNA-Seq Kit(Illumina)（NR103）+TIANSeq rRNA Depletion Kit（H/M/R) (NR101-TA) | NR103+NR101-TA |
| lncRNA | human cell line | lib-T2-TGF-TG | TIANGEN Biotech(Beijing)Co.,Ltd,China | TIANSeq Stranded RNA-Seq Kit(Illumina)（NR103）+TIANSeq rRNA Depletion Kit（H/M/R) (NR101-TA) | NR103+NR101-TA |

Table S1 library construction kits used in the study.
